# Host-Specific Serum Factors Control the Development and Survival of *Schistosoma mansoni*

**DOI:** 10.3389/fimmu.2021.635622

**Published:** 2021-04-23

**Authors:** Anisuzzaman, Sören Frahm, Ulrich Fabien Prodjinotho, Sonakshi Bhattacharjee, Admar Verschoor, Clarissa Prazeres da Costa

**Affiliations:** ^1^ Institute for Medical Microbiology, Immunology and Hygiene, Technical University of Munich (TUM), Munich, Germany; ^2^ Department of Parasitology, Bangladesh Agricultural University, Mymensingh, Bangladesh; ^3^ Department of Infectious Diseases and Microbiology, University of Lübeck, Lübeck, Germany; ^4^ Centre for Global Health, Technical University of Munich (TUM), Munich, Germany

**Keywords:** *Schistosoma mansoni*, host specificity, newly transformed schistosomula, host serum, schistomicidal activity, complement system, antibodies

## Abstract

**Introduction:**

Schistosomiasis is a neglected tropical disease (NTD) caused by blood-dwelling flatworms which develop from skin-penetrating cercariae, the freely swimming water-borne infective stage of *Schistosoma mansoni*, into adult worms. This natural course of infection can be mimicked in experimental mouse models of schistosomiasis. However, only a maximum of 20-30% of penetrated cercariae mature into fecund adults. The reasons for this are unknown but could potentially involve soluble factors of the innate immune system, such as complement factors and preexisting, natural antibodies.

**Materials and Methods:**

Using our recently developed novel serum- and cell-free *in vitro* culture system for newly transformed schistosomula (NTS), which supports long-term larval survival, we investigated the effects of mouse serum and its major soluble complement factors C1q, C3, C4 as well as preexisting, natural IgM *in vitro* and assessed worm development *in vivo* by infecting complement and soluble (s)IgM-deficient animals.

**Results:**

In contrast to sera from humans and a broad variety of mammalian species, serum from mice, surprisingly, killed parasites already at skin stage *in vitro*. Interestingly, the most efficient killing component(s) were heat-labile but did not include important members of the perhaps best known family of heat-labile serum factors, the complement system, nor consisted of complement-activating natural immunoglobulins. Infection of complement C1q and sIgM-deficient mice with *S. mansoni* as well as *in vitro* tests with sera from mice deficient in C3 and C4 revealed no major role for these soluble factors *in vivo* in regard to parasite maturation, fecundity and associated immunopathology. Rather, the reduction of parasite maturation from cercariae to adult worms was comparable to wild-type mice.

**Conclusion:**

This study reveals that not yet identified heat-labile serum factors are major selective determinants of the host-specificity of schistosomiasis, by directly controlling schistosomal development and survival.

## Introduction

Schistosomiasis, caused by blood-vessel dwelling trematodes (blood flukes), is a common parasitic disease and considered a neglected tropical disease (NTD). *Schistosoma mansoni*, the pathogen causing devastating intestinal schistosomiasis, is prevalent in Africa, the Middle East and South America (1, 2). More than 250 million people are infected, especially in tropical and subtropical areas, and 700 million, mostly children, are at risk (3). Thousands of people die each year (4) and several hundred millions are struggling with post-treatment residual morbidity (5). In the regions with typical transmission pattern, 60-80% of school-aged children and 20-40% of adults can remain actively infected (6) despite mass drug administration (MDA) campaigns. Annual loss of disease-adjusted life years (DALYs) is estimated at 2.54 million, the second most devastating parasitic disease after malaria (7), and recently the overall disease burden has increased further (8). Although the main anthelmintic treatment, praziquantel, is widely available and its administration rather simple, development of resistances and high reinfection rates limit its overall effectiveness (9, 10). In fact, praziquantel targets the adult worm, but has little or no effect on its preceding larval stages. Neither does praziquantel target parasite eggs, the main drivers of the immunopathology, granuloma-formation, and fibrosis mainly in the liver and intestinal tract, which is the cause of schistosomiasis-associated mortality and morbidity (2). Still, for new drug discovery, only newly transformed schistosomula (NTS) or *in vivo* generated adult worms have so far been available. Therefore, we recently developed a novel *in vitro* method that enables the development of other stages (e.g., lung stage, early liver stage and late liver stage (juvenile worms)), allowing the study of more stage-specific effects and potentiating effective new drug discovery (11).

Infection of the human host starts when *S. mansoni* cercariae, the free-living larvae that are released into fresh water by infected snails infect humans by penetrating intact skin and transforming into the succeeding developmental stage, the schistosomulae. From the moment schistosomulae leave the skin to enter the vasculature, all subsequent developmental stages (lung-stage schistosomulae (LuS), early liver-stage schistosomulae (eLiS), juvenile/late liver-stage schistosomulae (lLiS) and adult worms) (11) of the parasite take place in intimate contact with the host blood. Thriving in this hostile environment is a remarkable feature, as besides cells of the innate and adaptive immune system, the blood contains a vast array of highly effective defensive humoral serum factors, including complement factors and polyreactive natural antibodies that are mostly of the IgM isotype. Indeed, during its co-evolutionary development with its host, the parasite developed strategies to purposefully counteract serum proteins (e.g. binding and inhibiting complement pathways and antibodies (12–14). In mice, widely used to investigate various aspects of schistosomiasis, closer scrutiny of results suggests that serum factors may dominate the apparent inborn resistance to schistosomiasis: first, only ~30% of penetrating cercariae mature in the mouse (15), and, second, full-body irradiation or genetic ablation of *RAG1*, suppressing cellular immunity, does not notably improve *S. mansoni* maturation in mice (16, 17). Such observations support the notion that not yet identified serum components, rather than cellular factors, dominate the early murine resistance to invading cercariae.

In this study, we explore to what extent host-serum factors influence and contribute to schistosome development and survival. We report here that, in contrast to human serum, which in fact promotes and supports NTS survival and development *in vitro*, mouse serum rapidly killed NTS, revealing the presence of strongly schistomicidal serum compound(s) in murine blood. Furthermore, although the highly efficient schistomicidal component(s) of mouse serum was clearly heat-labile, it could not be attributed to the complement system nor preexisting natural IgM immunoglobulins. In addition, *in vivo* infection experiments using mice lacking selected antibody isotypes or key complement factors did not proceed significantly different from experiments using wild-type mice, supporting the notion that the mouse-specific schistomicidal serum activity must be derived from distinct therapeutic candidate compounds other than antibodies and three of the most abundant complement proteins in the serum including the central factor C3.

## Materials and Methods

### *S. mansoni* Life Cycle Maintenance

A Brazilian strain of *S. mansoni*, maintained in *Biomphalaria glabrata* snails as previously described (18, 19), was used in all experiments.

### Generation of NTS

NTS were generated as previously described (11). Briefly, cercariae were harvested from infected snails using the light induction method. After thorough washing, cercariae were resuspended in ice-cold HBSS medium (Cat. No. H6648, Sigma-Aldrich, Germany) supplemented with 200 U/ml Penicillin and 200 μg/ml Streptomycin (Cat. No. P4333, Sigma-Aldrich, Germany), pipetted vigorously 40 times, and then vortexed for three minutes at the highest speed to trigger tail loss, which was confirmed by microscopy (10x). Lost tails were removed by washing extensively with ice-cold HBSS. NTS were then re-suspended in culture media and counted.

### Animals and Sera

C57BL/6 mice were purchased (Envigo, Germany) or bred in-house. *C1q^-/-^*, *sIgM^-/-^*, *C3^-/-^*, *C4^-/-^* or *Rag1^-/-^* (C57BL/6 background) were bred in-house as described (20–24). Animals of both sexes were used. *In vivo* experiments and serum collection from all animals were approved and conducted in accordance with local government authorities Bezirksregierung Oberbayern (license number AZ 55.2-1-54-2532-115-14). Sera from horses, swine, sheep, hamsters, rabbits and rats were prepared from blood collected by venipuncture in non-medicated Falcon tubes from well-restrained anaesthetized horses, swine, bovines, sheep or rabbits. Hamsters, rats and mice were euthanized before serum preparation.

Animals were maintained under specific pathogen-free conditions at the Institute for Medical Microbiology, Immunology and Hygiene (MIH) and at the Center for Preclinical Research (CPR) (Technical University of Munich (TUM)) in accordance with national and EU guidelines 86/809.

### Origin and Preparation of Human and Non-human Primate Serum

Sera were prepared from fresh blood collected from *S. mansoni*-naïve non-human primates (NHP, rhesus macaques) (license number AZ 33.9-42502-04-12/0704, Deutsches Primatenzentrum, Göttingen, Germany) and from consenting healthy volunteers with no previous history of schistosomiasis as approved by the TUM ethical committee (license number AZ 215/18 S). Fresh blood was left to clot at room temperature for 30 min., then centrifuged at 1845 g for 20 min and serum was collected and stored at -20°C until further use. Sera were collected from both male and female individuals and pooled before further use. For bovine serum, commercially available FCS (Sigma, Germany) was used.

### NTS Assays With Sera From Different Hosts

To compare the effects of the serum-specific factors of human and mouse, the main definitive and laboratory host, respectively, we cultured NTS (100 NTS in 150 µl) in a 96-well flat bottom tissue culture plate (Cat. No. 353075, Corning Incorporated, USA) in hybridoma medium (HM, HybridoMed Diff 1000, Biochrom GmbH, Germany) supplemented with 200 U/ml Penicillin and 200 μg/ml Streptomycin, adding their serum at different dilutions (0-40%) and scored at day seven. The NTS were incubated at 37°C in 5% CO_2_ and humidified air. Additionally, NTS were cultured following the same procedure in the presence or absence of 20% human or mouse sera and scored initially at day zero, one and three and then weekly up to four weeks. Medium was replaced weekly. To determine the host specificity and observe developmental changes, we cultured and maintained NTS in hybridoma medium (100 NTS in 150 µl) supplemented with 200 U/ml Penicillin and 200 μg/ml Streptomycin with or without 20% serum derived from NHP, horses, swine, bovines, sheep, hamsters, rabbits, or rats for four weeks. Stage determination was assessed visually using an inverted microscope (10x) (Zeiss, Germany). The skin stage (SkS) presented with a plump almost oval shape and irregular contractions, the lung stage (LuS) presented with an initial elongation and increase in activity/contractions, the early liver stage (eLiS) presented itself with a clearly visible bifurcated gut as well as a drastic increase in overall size. The juvenile worm stage (lLiS) showed a growing elongation of the aboral part of the body as well as further differentiation of the oral and ventral sucker. For heat inactivation, mouse serum was treated at 56°C for 30 min. NTS were maintained in hybridoma medium supplemented with 200 U/ml Penicillin and 200 μg/ml Streptomycin (100 NTS in 150 µl) adding 20% mouse serum with or without heat inactivation and scored accordingly. Furthermore, NTS were cultured and maintained in hybridoma medium supplemented with 200 U/ml Penicillin and 200 μg/ml Streptomycin (100 NTS in 150 µl) with 20% mouse serum derived from *C1q* ^-/-^, s*IgM^-/-^*, *C3^-/-^*, *C4^-/-^, Rag1^-/-^* or wild-type C57BL/6 mice, and viability was scored at the indicated time points. Finally, NTS were cultured in media supplemented simultaneously with both human and mouse sera at different concentrations (10-20%). In all experiments, all conditions were carried out in technical triplicates. All experiments were repeated at least three times.

### Viability Scoring of NTS

Viability scoring was performed visually using an inverted microscope (10x) (Zeiss, Germany) as previously described (11). Briefly, the scoring was assessed as an average across all parasites per well and ranged from zero (no movement, heavy granulation, blurred outline, rough outer tegument and blebs) to one (strongly reduced motility, rough outer tegument and blebs), to two (reduced motility or increased uncoordinated activity, slight granulation, intact tegument with slight deformations) to three (regular smooth contractions, no blebs and smooth outer surface, no granulation with clear view of internal structures) (**Supplementary Table 1**). After mechanical transformation and before adding serum, each well of parasites was scored microscopically based on the scoring system described in **Supplementary Table 1**. To ensure no excessive transformational damage, investigation was only continued if a score of two or higher was reached. Due to applying the score as an average it was applied in 0.25 steps per well. Experiments were repeated three times with technical triplicates for each condition. Each data point is presented here as mean ± SD with pooled data from all repeat experiments (n=3). For determination of larval development stage, morphological characteristics were used as described (11).

### Infection of *sIgM^-/-^* or *C1q^-/-^* Mice and Assessment of Parasite Maturation and Fecundity

To assess the maturation and fecundity of parasites, wild-type (WT), s*IgM^-/-^* or *C1q^-/-^* mice were infected by injecting subcutaneously 200 viable cercariae. Each experiment was repeated at least four times (WT, n=28; *sIgM^-/-^*, n=12; *C1q^-/-^*, n=34). Survival of animals was monitored up to 11 weeks. At indicated time points, animals were euthanized, and mature worms were flushed out of the portal vein to determine worm burden, size and gender of the worms. Additionally, liver egg burden was analyzed as described before (18, 25). Briefly, livers were collected from infected, euthanized animals as mentioned above at indicated time points minced and digested with KOH for 2 h at 37°C temperature under continuous shaking. Digested tissue was centrifuged at 1500 rpm for 10 min and vortexed before counting under a microscope.

### Histopathology

To estimate the size of granuloma, WT, s*IgM^-/-^* or *C1q^-/-^* mice were infected by injecting subcutaneously 200 viable cercariae as mentioned above and livers were collected. Livers were preserved in 10% buffer neutral formalin and washed with phosphate buffer saline (PBS) and embedded in paraffin. Thin sections (4 µm) were prepared and stained with Masson’s Blue. At least three sections from each sample were evaluated to estimate the size of granuloma. Average granuloma size from individual mice was calculated by assessing 30-40 granulomas/section. The experiment was repeated at least four times.

### ELISA

WT or *C1q^-/-^* mice were infected by injecting subcutaneously 200 viable cercariae as mentioned above and lymphocytes from mesenteric lymph nodes (MLN) were collected. Bulk lymphocytes (2x10^5^) from non-infected WT, infected WT or C1q^-/-^ mice were cultured in RPMI medium at 37°C and 5% CO_2_ in humidified air and were stimulated *in vitro* with soluble egg antigen (SEA) (20 µg ml^-1^) or anti-CD3/28 (Miltenyibiotec.com, Germany) (1 µg ml^-1^) for 48 h. IFN-γ and IL-10 levels were analyzed in the culture supernatants by ELISA (Ready-SET-Go!, eBioscience, USA) following the manufacturer’s instructions. Each experiment was repeated at least three times (total mouse number WT, n=28; C1q^-/-^, n=34). Each condition was carried out as technical triplicates. SEA was prepared as described before (25, 26).

### Quantification and Statistical Analysis

Data were presented as mean ± SD for multiple group comparisons. One-way ANOVA followed by post-hoc Bonferroni’s analysis was used for normally distributed data, otherwise it was followed by post-hoc Kruskal-Wallis’ analysis. For direct comparisons, unpaired two-tailed Student’s *t-*test was employed if normally distributed otherwise a Mann-Whitney-test was employed. A value of *P* < 0.05 was considered as significant.

## Results

### Murine Serum Rapidly Kills Whereas Human Serum Supports Larval Development

**Figure 1:**
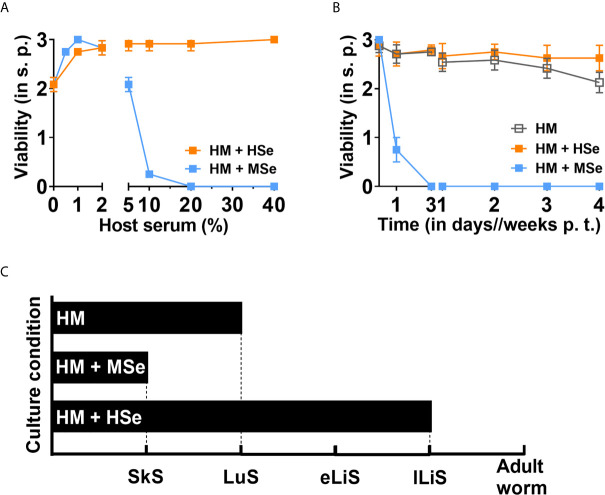
Mouse serum rapidly kills NTS whereas human serum promotes their development and survival. NTS were cultured and maintained in hybridoma medium (HM) with or without host sera at 37°C in 5% CO_2_ for four weeks. Medium was refreshed weekly. **(A)** Mouse serum (MSe) kills NTS in a concentration-dependent manner. NTS were cultured in the absence or presence of increasing concentrations of human serum (HSe) or MSe and the viability scored at day seven. **(B)** MSe kills NTS rapidly. NTS were cultured in HM in the absence or presence of 20% of HSe or MSe and viability scored at indicated time points. Results are representative of at least three individual experiments. Each data point has been shown as mean ± SD of at least three technical replicates. **(C)** Schematic presentation of development of schistosomula. NTS were cultured in HM in the presence or absence of HSe or MSe in the same manner and monitored as above and the development assessed. SkS, skin stage; LuS, lung stage; eLiS, early liver stage; lLiS, late liver stage; p.t., post transformation; s.p., scoring point.

After entering their definite host, schistosomes migrate and develop in a hostile and immune defense-rich environment, including the skin, blood, lungs and lymphatic system, which all contain soluble serum factors to different extents. Human is the primary definitive host for *S. mansoni*, and mouse is the most widely used experimental laboratory host. Previous research suggests that in various mouse strains only a maximum of 30% of all penetrated cercariae mature to adult worms (15). It was proven that this is at least partly due to soluble (humoral) serum factors rather than cellular immunity (15–17). Our recently established *in vitro* serum- and cell- free long-term culture system allowed us to test the effect of host-specific serum factors (11). We observed that HM supplemented with 20% of human serum (HSe) supported the larval development and survival *in vitro* up to the juvenile worms (lLiS), the pre-pairing stage of *S. mansoni* (**Figures 1A–C**) from skin-stage schistosomulae (SkS) *via* lung stage (LuS) and early liver stage (eLiS). To our surprise, we found that mouse serum (MSe) efficiently killed all NTS in a concentration-dependent manner (**Figure 1A**). Specifically, MSe concentrations higher than 10% killed all NTS by day three (**Figure 1B**). In contrast, HSe did not kill NTS at any concentration tested (1-40%) but rather supported the larval survival and development *in vitro* throughout the entire culture period (minimum of four weeks) with a viability score nearing three (**Figures 1A, B**). In addition, although larval survival was supported in the presence of HM alone, the development of the NTS was halted at the lung stage (**Figure 1C**). Importantly, this developmental halt in the serum-free condition was overcome by adding 20% HSe, promoting development to the juvenile worms, the pre-pairing stage of *S. mansoni* (**Figure 1C**). Thus, in contrast to HSe, MSe harbors non-permissive factors for schistosomal development and survival.

### Host Serum Dictates Larval Survival and Development

**Figure 2:**
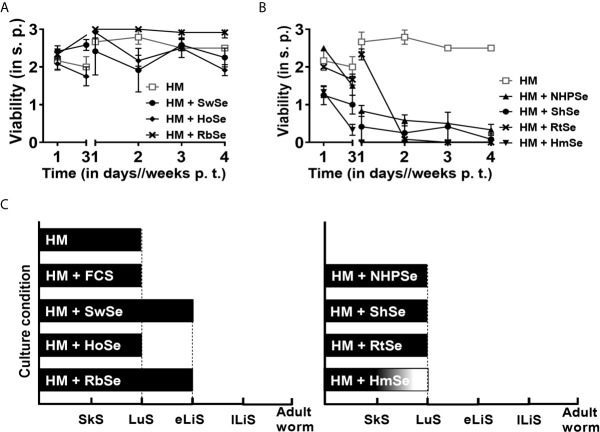
Short- and long-term survival of NTS in serum is host specific. NTS were cultured and maintained in hybridoma medium (HM) with or without 20% mammalian sera at 37°C in 5% CO_2_ for four weeks. Medium supplemented with 20% of the corresponding serum was refreshed weekly. **(A)** Serum effects of mammalian species which ensured long-term survival of NTS. NTS were cultured in the absence (HM) or presence of 20% serum from swine (SwSe), horses (HoSe) or rabbits (RbSe) and viability was scored. All sera ensured survival of NTS for at least four weeks after transformation. **(B)** Serum effects of mammalian species that heavily impaired viability of NTS. NTS were cultured in the absence or presence of 20% serum from rhesus macaques (NHPSe), sheep (ShSe), rats (RtSe) or hamsters (HmSe) and viability was scored. Results are representative of at least three individual experiments. Each data point has been shown as mean ± SD of at least three technical replicates. **(C)** Schematic representation of stage-dependent development of schistosomula. NTS were cultured with sera of indicated species in the same manner as above and monitored microscopically. SkS, skin stage. LuS, lung stage. eLiS, early liver stage. lLiS, late liver stage. p.t., post transformation. s.p., scoring point.

Since mouse and human sera influenced the survival and development of NTS in such a contrasting manner, we sought to unveil whether those effects were exclusive to humans and mice or if sera from other mammalian species also exert similar effects. To evaluate long-term survival and development of NTS, we cultured NTS in HM in presence or absence of serum from schistosome-naïve rhesus macaques as a non-human primate (NHPSe), horses (HoSe), swine (SwSe), bovines (FCS), sheep (ShSe), rabbits (RbSe), hamsters (HmSe) or rats (RtSe) at the same concentration (20%) as before. NTS survived well in the culture media supplemented with RbSe, with a viability score of 2.92 ± 0.14 after four weeks of culture comparable to that in HSe (**Figures 2A, B**). However, in the presence of RbSe, NTS developed only up to the eLiS and not to juvenile worms like in HSe (**Figure 2C**). NTS also survived well in both swine (2.25 ± 0.25) and horse (1.92 ± 0.14) sera (**Figure 2A**), but again never reached the eLiS (**Figure 2C**). In contrast, viability of NTS cultured in HM supplemented with serum from NHP, evolutionarily most related to human but not a natural host of *S. mansoni*, started to decline quickly within the first three days and very few NTS survived until week four (**Figure 2B**) and again their development stagnated in the lung stage similarly to ShSe, RtSe and HmSe, which, however, killed nearly all NTS by week two of culture (**Figures 2B, C**) but not as effectively as MSe. This prompted us to characterize the properties of the components with killing effect in mouse serum.

### Both Heat-Stable and Heat-Labile Mouse Serum Components Contribute to Killing of *S. mansoni*

**Figure 3:**
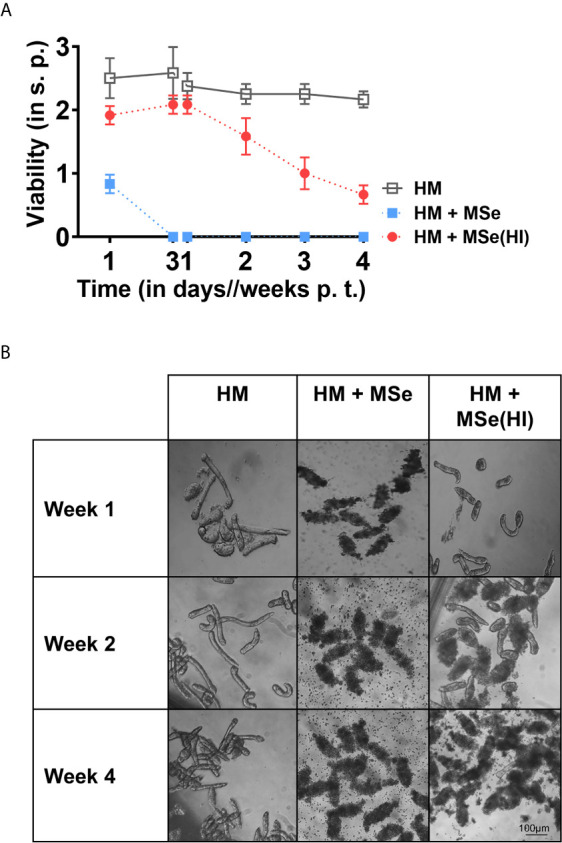
Rapid killing effects of mouse serum are mainly due to heat labile factors. NTS were cultured in hybridoma medium (HM) supplemented or not with 20% of native (MSe) or heat-inactivated (56°C for 30 min) (MSe(HI)) mouse sera at 37°C in 5% CO_2_ for four weeks. Supplemented medium was refreshed weekly. HM alone was used as a control. **(A)** Survival of NTS is prolonged by heat inactivation of serum. Viability scoring was performed at the indicated time points. Results are representative of at least three individual experiments. Each data point has been shown as mean ± SD of at least three technical replicates. **(B)** Morphological changes of NTS induced by MSe with and without heat inactivation. Representative photographs of NTS were taken from the NTS cultured using a digital camera fitted with an inverted microscope (10x). p.t., post transformation; s.p., scoring point.

Components of the complement system are crucial for innate and adaptive immunity (27). Therefore, we investigated whether the NTS killing capacity of MSe could be abrogated by heat inactivation since those components are sensitive to heat treatment. Interestingly, we observed that heat-inactivated MSe (MSe(HI)) abrogated the killing effect at day three as viability of NTS is clearly improved (2.10 ± 0.14) compared to non-inactivated MSe, in presence of which all NTS were already dead (0 ± 0) (**Figure 3A**). Within the first seven days of culture, NTS survived equally well in medium supplemented with MSe(HI) as in the control HM (**Figures 3A, B**). Still, even though NTS initially survived well in the presence of heat-inactivated serum within the first week of transformation, survival rapidly declined thereafter, with only few larvae surviving after two or four weeks (viability of 0.67 ± 0.14) (**Figures 3A, B**). Taken together, our results show that heat-labile MSe factors strongly contribute to rapid killing effects of MSe, and that heat-stable factors affect long-term survival of NTS.

### Natural Murine Immunoglobulins Are Not Involved in Larval Killing

**Figure 4:**
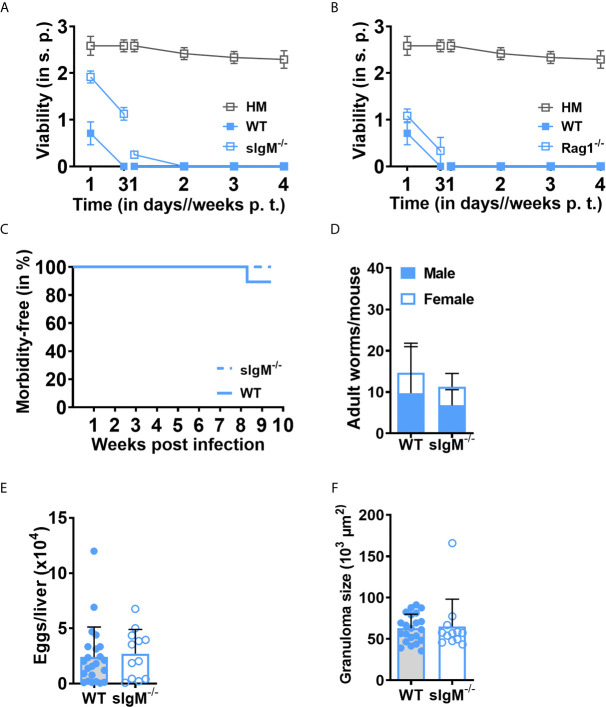
Unspecific immunoglobulins (Igs) have no impact on the viability of *S. mansoni* NTS. NTS were cultured and maintained using hybridoma medium (HM) supplemented with 20% *sIgM^-/-^*, *Rag1^-/-^*, or wild-type mouse (WT) sera at 37°C in 5% CO_2_ for four weeks. HM alone was used as a control. **(A)** Viability of NTS is not restored by the loss of soluble IgM (sIgM) or **(B)** Igs. Results are representative of at least three individual experiments. Each data point has been shown as mean ± SD of at least three technical replicates. **(C)** Loss of sIgM does not significantly influence the mortality of *S. mansoni*-infected mice. sIgM-deficient mice were infected by injecting 200 cercariae and survival of the animals was monitored on a weekly basis. **(D)** Worm maturation was not affected by the deficiency of sIgM. After ten weeks of infection, the animals were euthanized and mature worms from mesenteric veins were flushed out, enumerated and male/female ratio was determined. Shown data is the mean ± SD of worms per mouse. **(E)** Lack of sIgM does not affect the fecundity of the worm. Eggs from the liver were isolated and counted. **(F)** sIgM does not influence the egg-induced immunopathology of the worm. Liver sections (4 µm) from infected wild-type or *sIgM^-/-^* mice were stained with Masson’s Blue and the diameter of 30-40 granulomas/section was measured microscopically (10x). Data shown is pooled data from five individual experiments (WT, n=28; sIgM^-/-^, n=12). Each data point shows a single mouse. Mean ± SD is indicated with bars. p.t., post transformation. s.p., scoring point.

Besides a heat-labile murine serum component that induced rapid larval killing, we also identified the existence of a slower acting, heat-stable serum compound, which contributed to the killing effect of MSe. As outlined earlier, *Schistosoma* has been known to bind antibodies (14), presumably in a fashion that neutralizes their immune effector function. As antibodies are comparatively heat stable, we tested their contribution to the killing of NTS *in vitro* by using sera from completely antibody deficient *Rag1^-/-^* mice or soluble IgM deficient (s*IgM* ^-/-^) mice. We observed that *sIgM^-/-^* and *Rag1^-/-^* sera already significantly affected NTS viability at day three and killed NTS in the same manner by day seven (*sIgM^-/-^* (0.25 ± 0), *Rag1^-/-^*(0 ± 0)) compared to wild-type (WT) (0 ± 0) serum, ruling out the possibility of lethal effects by natural IgM (**Figure 4A**) and immunoglobulins in general (**Figure 4B** and **Supplementary Figure 1**). We next sought to verify whether antibodies indeed have a subordinate role in the control of *Schistosoma* in an *in vivo* setting. We tested this in the *sIgM^-/-^* mouse for several reasons: first, IgM is the most potent complement activating antibody isotype. Second, it is the first antibody isotype to emerge upon B cell activation, before class switching to IgG and further isotypes. Third, “natural” IgM, with comparatively high avidity to conserved microbial compounds, is present even in naïve sera and has the potential to target NTS upon first contact with blood. Finally, unlike *Rag1^-/-^* mice, *sIgM^-/-^* mice have polyclonal B and T cell compartments that ensure a normal secondary lymphoid organ architecture (28). Still, we did not observe any significant difference between *sIgM^-/-^* and WT mice (**Figures 4C–F**). Infected sIgM-deficient mice tolerated the infection compared to WT mice, survived well up to euthanizing (ten weeks post infection) (**Figure 4C**) and showed comparable worm burden and male/female ratios (**Figure 4D**). Also, no significant difference in liver egg burden was observed (**Figure 4E**), implying that fecundity of the worm was not influenced by the loss of IgM. Finally, the development of liver granuloma was similar in *sIgM^-/-^* and WT mice (**Figure 4F**). Taken together, these data strongly indicate that antibodies present in MSe do not significantly influence the survival and maturation of *Schistosoma*.

### Complement C1q, C3 and C4 Proteins Have No Role in Larval Killing by Murine Serum

**Figure 5:**
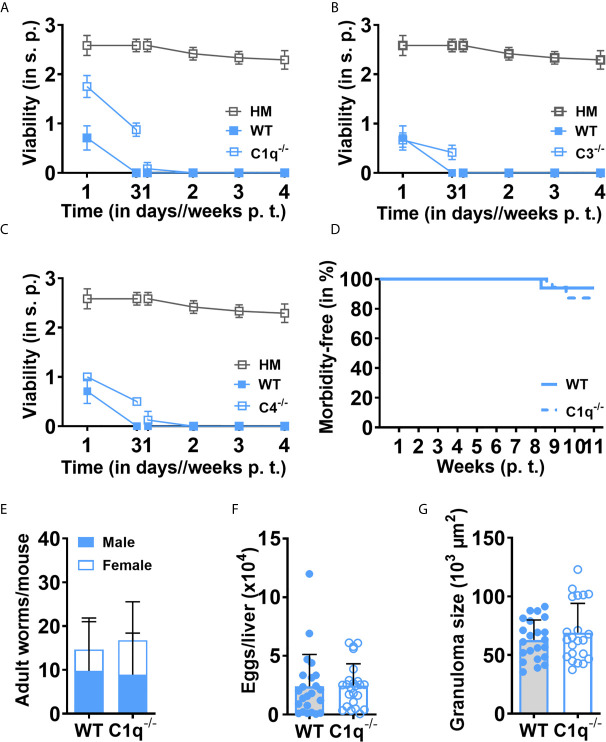
Loss of C1q alters neither rapid killing of NTS nor *in vivo* maturation and immunopathology after *S. mansoni* infection. NTS were cultured and maintained using sera collected from **(A)** *C1q^-/-^*, **(B)** *C3^-/-^*, **(C)** *C4^-/-^* or wild-type (WT) mice and viability scoring was performed at the indicated time points. Hybridoma medium (HM) alone was used as a control. Results are representative of at least three individual experiments. Each data point has been shown as mean ± SD of at least three technical replicates. **(D)** Loss of C1q does not significantly influence the mortality of *S. mansoni*-infected mice. C1q-deficient mice were infected by injecting 200 cercariae subcutaneously and survival of the animals was monitored on a weekly basis. **(E)** Worm maturation was not affected by the deficiency of C1q. After nine to 11 weeks of infection, the animals were euthanized and mature worms from mesenteric veins were flushed out, enumerated and male/female ratio was determined. **(F)** Lack of C1q does not affect the fecundity of the worm. Animals were infected and euthanized, and eggs from a weighted liver were isolated and counted. **(G)** C1q does not influence the egg-induced immunopathology of the worm. Liver sections (4 µm) from infected wild-type or *C1q^-/-^* mice were stained with Masson’s Blue and the diameter of 30-40 granuloma/section was measured under microscope (10x). Data shown is pooled data from five individual experiments (WT, n=28; *C1q^-/-^*, n=34). Each data point shows a single mouse. Mean ± SD is indicated with bars. p. t., post transformation; s.p., scoring point.

The complement system is a prototypic example of a heat-labile cascade of zymogens (29, 30). We, therefore, considered that the rapid, heat-labile killing of schistosomulae could be due to the complement system, a heterogeneous group of more than 20 proteins circulating in the blood, with C1q, C3 and C4 playing key roles in the initiation and/or sustenance of the three main complement activation pathways (classical, lectin, and alternative). We thus tested the role of murine C1q, C3 or C4 by comparing the effects of sera from *C1q^-/-^, C3^-/-^, C4^-/-^* or WT mice on the survival of NTS. Against our expectations, no obvious improvement in NTS viability was observed in the absence of C1q (**Figure 5A**), C3 (**Figure 5B**) or C4 (**Figure 5C**). Indeed, all NTS died at day seven in presence of complement-deficient sera as in WT serum, suggesting that the tested complement factors, which control all characterized complement activation pathways, are not directly or dominantly involved in the rapid killing of NTS by the heat-labile murine serum compound(s) that we observed *in vitro*. We further verified if these *in vitro* observations would be equally reflected in the *in vivo* infection setting. Indeed, C1q-deficient mice showed no significant difference in survival compared to WT mice (**Figure 5D**). *C1q^-/-^* and WT mice also displayed comparable adult worm burden and development, and male and female ratio (**Figure 5E**). Eventually, assessment of classical parameters of schistosome-induced immunopathology, such as liver egg count (**Figure 5F**) and granuloma size (**Figure 5G**), did not display significant differences. In addition, schistosome egg-specific immune response as determined by SEA-induced IFN-γ (**Supplementary Figure 2A**) and IL-10 (**Supplementary Figure 2B**) secretion by splenocytes was comparable between WT and C1q-deficient mice. These results imply that the most abundant heat-labile complement components, such as C1q, C3 and C4, affecting all three complement activation pathways are not the compound(s) displaying prominent schistomicidal activity in murine serum.

### Murine Schistosomicidal Activity Is Preserved in Human Serum

**Figure 6:**
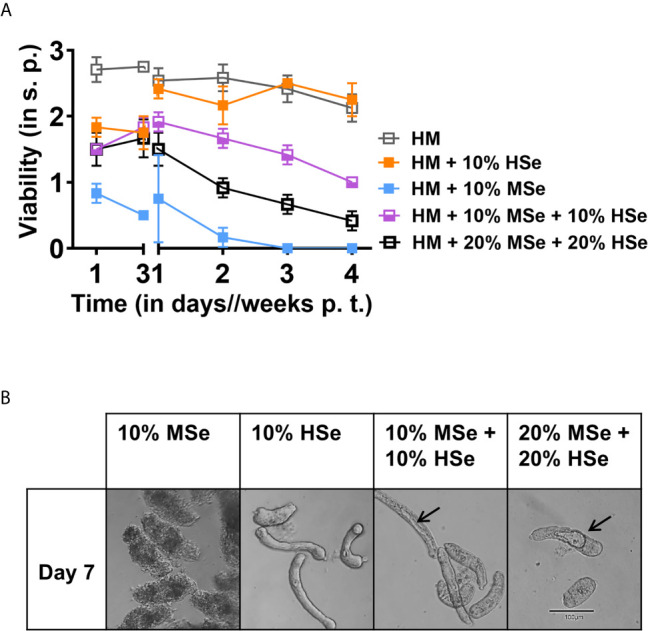
Mouse serum factors can partially antagonize survival promoting effects of human serum. **(A)** NTS were cultured and maintained in the presence of 10% of mouse (MSe) or human (HSe) sera or adding both at 10-20% and scoring was performed at the indicated time points. Hybridoma medium (HM) alone was used as a control. Each data point has been shown as mean ± SD of at least three technical replicates. **(B)** Morphological changes of NTS in presence of 10% MSe, HSe or in combination (10-20%). Representative photographs of NTS were taken using a digital camera fitted with an inverted microscope (10x). Arrow indicates development of vacuoles. p.t., post transformation; s.p., scoring point.

Given the lack of therapeutic agents that target the different larval developmental stages of *Schistosoma*, we tested whether the schistosomicidal activity of mouse serum is preserved in the presence of HSe, a prerequisite for any therapeutic potential. To do so, we incubated NTS in HM supplemented with 10-20% of mouse and 10-20% of human sera. Strikingly, even in the presence of HSe, which, previously, strongly promoted larval developmental and survival, addition of 10-20% MSe killed NTS overtime (**Figure 6A**). The killing effect is more pronounced in presence of 20% MSe. We observed that viability peaked at 1.50 ± 0.20 at day seven and afterwards gradually declined to 0.42 ± 0.10 at week four of culture. Morphologically, although the tegument of the NTS seems less affected, distinct vacuoles developed in the gut of the larvae within seven days of culture with increased cytoplasmic granularity in contrast to NTS maintained in HSe alone (**Figure 6B**), suggesting that internal organs may possibly be more vulnerable to the killing effects of MSe than the outer surface of the larvae. Importantly, the results indicate that murine schistosomicidal activity is retained in the presence of HSe, opening the door to further characterization and isolation of the compound(s) for potential therapeutic use in humans.

## Discussion

Despite decades-long MDA with anthelminthics, mostly praziquantel, the size of the schistosome infected population has not decreased substantially but has rather increased, resulting in severe socioeconomic problems in the endemic countries (1, 8, 31). Efforts to develop effective control strategies, such as a therapeutic agent targeting all mammalian, host-dwelling stages or a protective vaccine, have increased. But, to rapidly and efficiently screen a large number of new drugs, an easy, reliable and cost-effective *in vitro* culture technique is essential that supports long-term survival and development of the different stages of the parasite. We have developed a novel, reliable and highly standardized *in vitro* serum and cell-free method which ensure to test specific effects of potential candidate components on early and advanced larval stages, phenotypically comparable to most previously published works for *ex vivo* harvested parasites (11, 32).

Currently, the most widely used preclinical animal model for *in vivo* schistosome infection studies and for maintenance of the lifecycle under laboratory condition, is the mouse. It allows the establishment of patency and mimics most of the immunopathologies observed in humans. However, this is achieved at the cost of a surprisingly high loss of invading cercariae of up to 70%. In this line, we show here that mouse serum added to hybridoma medium killed NTS very rapidly within three days, even though hybridoma medium on its own supports NTS survival for at least four weeks. This finding clearly suggests that massive death of NTS in the presence of mouse serum is not merely due to nutrient deficiency present in mouse serum but rather argues for the presence of a component(s), which actively kill(s) the larvae. This notion of potentially harmful soluble factors contained within mice is further supported by the finding that, *in vivo*, death of a large number of penetrants still occurs in completely immunodeficient, whole-body irradiated mice (15, 16). In sharp contrast, hybridoma medium supplemented with human serum not only supported the prolonged survival of NTS but also promoted *in vitro* development of NTS up to juvenile worms, the pre-pairing adult stage. Up to recently, this has only been observed *in vitro* upon continuous feeding of larval stages with RBC (33–35), suggesting further the problematic nature of using mouse models as a ‘black-box test bed’ to study vaccine or therapeutic targets in schistosome-challenge infection or to test blood soluble factors such as complement factors.

The complement system has been shown to play a role in the killing of pathogens (36); however, hemoparasites, including schistosomes, have evolved diversified evasion strategies through co-evolutionary processes. These include shedding of the surface membrane glycocalyx by cercariae upon invasion, binding of antibodies and complement components or expression of molecules (e.g., paramyosin), which cleave complement factors (13, 36), indicating that complement and antibodies form a relevant challenge to the parasite to develop immune evasion strategies against them. Antibodies and complement are closely intertwined immune effector systems, with antibodies, especially antigen-bound IgM, being major initiators of the classical pathway of complement activation, providing a “docking point” for C1q, which then leads to C2, C4 and to C3 activation, the central component in which all three complement pathways, including the alternative pathway, converge for pathogen lysis (37–39). Although a study reported that C3-deficiency in schistosome-infected mice has no effect on worm development or liver pathology (40), the role of complement factors and antibodies in reducing the progression of schistosomes within the host remains elusive. Within our studies, we expand on these findings substantially and demonstrate, using gene-deficient mice, that upstream components like C1q or IgM did not affect the survival, development, fecundity or immunopathology of the worms. Furthermore, we show that neither C1q, C3, C4 nor antibodies were responsible for the killing effect of mouse serum on NTS. Furthermore, C2 and factor B, one of the most abundant serum complement proteins and key activators of the alternative pathway, are heat sensible (41, 42). Their influence as a major contributor to the killing effect could be ruled out by heat inactivation of serum. Additional investigations are required to fully elucidate the role of all the components of the complement system such as MBL-associated serine proteases (MASPs) (MBL/lectin pathway). Taken together, we conclude that the major complement proteins C1q, C3 and C4, key players in classical and alternative complement pathways, are not playing a major role in the immunological defense against penetrating *S. mansoni* and their development in mice.

To investigate further if the killing effect observed in mouse serum is unique, we applied our *in vitro* NTS culture system to study the effects of serum, harvested from a large number of mammalian species. The investigated species were phylogenetically diverse and included those which are commonly used in various laboratory procedures or models. Of these, surprisingly rabbit serum, which is phylogenetically closer to rodents, such as mice, rats and hamsters whose sera killed NTS, promoted NTS survival and development up to eLiS. Further development could not be observed, however. Interestingly though, rabbits are known to be non-permissive hosts in which development is stunted (43); furthermore, in rabbits, percentages of worms comparable to mice can be retrieved (44). Taken together, it seems that in schisto-naïve rabbits, soluble factors might only play a minor role. Interestingly, this could indicate two different selection time points for the determination of host specificity: Mice could be seen as an example for early mechanisms in defense whereas rabbits could be seen as an example for a later defense mechanism.

Another interesting finding within this study was the dramatic, detrimental effect on early NTS survival caused by serum from rhesus macaques, the closest relative to humans that we tested so far. In fact, we noticed that most NTS died rapidly but some single larvae survived until week four with a low viability score and reduced motility. Indeed, previous reports suggest that rhesus macaques have a strong ‘self-cure’ mechanism against both *S. mansoni* and *S. japonicum* (45, 46) whereby the exact mechanism driving this ‘self-cure’ mechanism is still unclear. About 43% of penetrated cercariae became mature in infected macaques (47) and the recovered parasites from infected macaques showed severe developmental defects with altered ultrastructural architecture (45). However, there still seems to be quite dramatic differences between different non-human primates; the rhesus macaque, for example, seems to be a rather poor host compared to the baboon (43). The only important naturally occurring final host for *S. mansoni* is the human (48, 49). This might explain why *S. mansoni* NTS not only survived but also developed to juvenile stage *in vitro* solely upon addition of serum from humans and not upon addition of sera from other tested species that supported long term survival and development. Importantly, the schistosomicidal activity of the killing component(s) in mouse serum is preserved in the presence of human serum at both lower and higher concentration, revealing a notable feature for any therapeutic potential. The schistosomulae-killing mechanism of mouse and other animal sera is yet to be revealed; however, the schistosomicidal component(s) present in the ‘killer group’ sera possibly inhibit lipid metabolism, thus preventing the synthesis of the cuticle of schistosomulae, which was evident by the development of blabbing and roughness of the tegument. Moreover, an important group of heat-labile factors in serum to focus on are enzymatic proteins and lipo-proteins, reported to exhibit anti-schistosome activity as well (50, 51). This may have relevance in our setting as the killing component(s) in mouse serum strongly affect the integrity of schistosome tegument, which is crucial for parasite survival. Additionally, massive granules (vacuoles) are developed in the severely devitalized or dead schistosomulae, indicating restriction of energy metabolism. However, our research is in progress to identify the killing component(s) present in the mouse sera as well as its mechanism.

Cumulatively, our data identify a new level of host-pathogen interaction in schistosome biology. We present here a contrasting effect of different host sera on schistosome survival and development. We reveal that not yet identified component(s) present in mouse, but not in human serum efficiently kill NTS. The component(s), which do not belong to the complement system or antibodies, are partially heat stable and heat labile. Revealing the identity of the culprit(s) responsible for the killing are important future tasks that will endorse our understanding of helminth-host crosstalk at the early developmental stage and might lead to the discovery of new drug candidates.

## Data Availability Statement

The original contributions presented in the study are included in the article/**Supplementary Material**. Further inquiries can be directed to the corresponding author.

## Ethics Statement

The study was approved by TUM ethical committee (license number AZ 215/18 S). The patients/participants provided their written informed consent to participate in this study. The animal study was reviewed and approved by Bavarian government authorities (Bezirksregierung Oberbayern) (license number AZ 55.2-1-54-2532-115-14).

## Author Contributions

Conceived and designed the research: CPC, AV, and A. Performed the experiments: A, SF, UFP, and SB. Processed and analyzed the data: SF, UFP, A, and CPC. Prepared the figures: A, SF, UFP, and CDC. Wrote the main manuscript: A, SF, UFP, and CPC with input from AV and SB. All authors reviewed the manuscript. All authors contributed to the article and approved the submitted version.

## Funding

Financial support was provided by DFG 1469/15-1. SF was supported by the doctoral program in Translational Medicine of the TUM School of medicine. A was supported by a postdoctoral fellowship for foreign researchers by the Alexander von Humboldt Foundation (Georg Foster Program).

## Conflict of Interest

The authors declare that the research was conducted in the absence of any commercial or financial relationships that could be construed as a potential conflict of interest.

## Supplementary Material

Supplementary Figure 1 Morphological effect of *Rag1^-/-^* serum on NTS. NTS were cultured in HM in the presence or absence of 20% WT or *Rag1^-/-^* serum. Microphotographs were taken at indicated time points with a digital camera fitted to an inverted microscope (10x).

Supplementary Figure 2 Loss of C1q does not alter cytokine production by splenocytes. Lymphocytes (2x10^5^) from wild-type or *C1q^-/-^* mice infected for 11 weeks with *S. mansoni* were re-stimulated *in vitro* with or without (Ctrl) SEA (20 µg/ml) or anti-CD3/28 (1µg/ml) for 48 h. Secreted levels of IFN-γ **(A)** or IL-10 **(B)** were analyzed in the culture supernatants by ELISA. Graph shows representative data from at least 3 independent infection experiments. Each data point has been shown as mean ± SD of at least three technical replicates. (*P <0.05, **P <0.01). Inf, infected.

Supplementary Table 1 Viability scoring points description.
