# Supplementary figures and images for: Host-Specific Serum Factors Control the Development and Survival of Schistosoma mansoni
Source: Front Immunol. 2021 Apr 23;12:635622. doi: 10.3389/fimmu.2021.635622 (PMC8103320; doi:10.3389/fimmu.2021.635622)

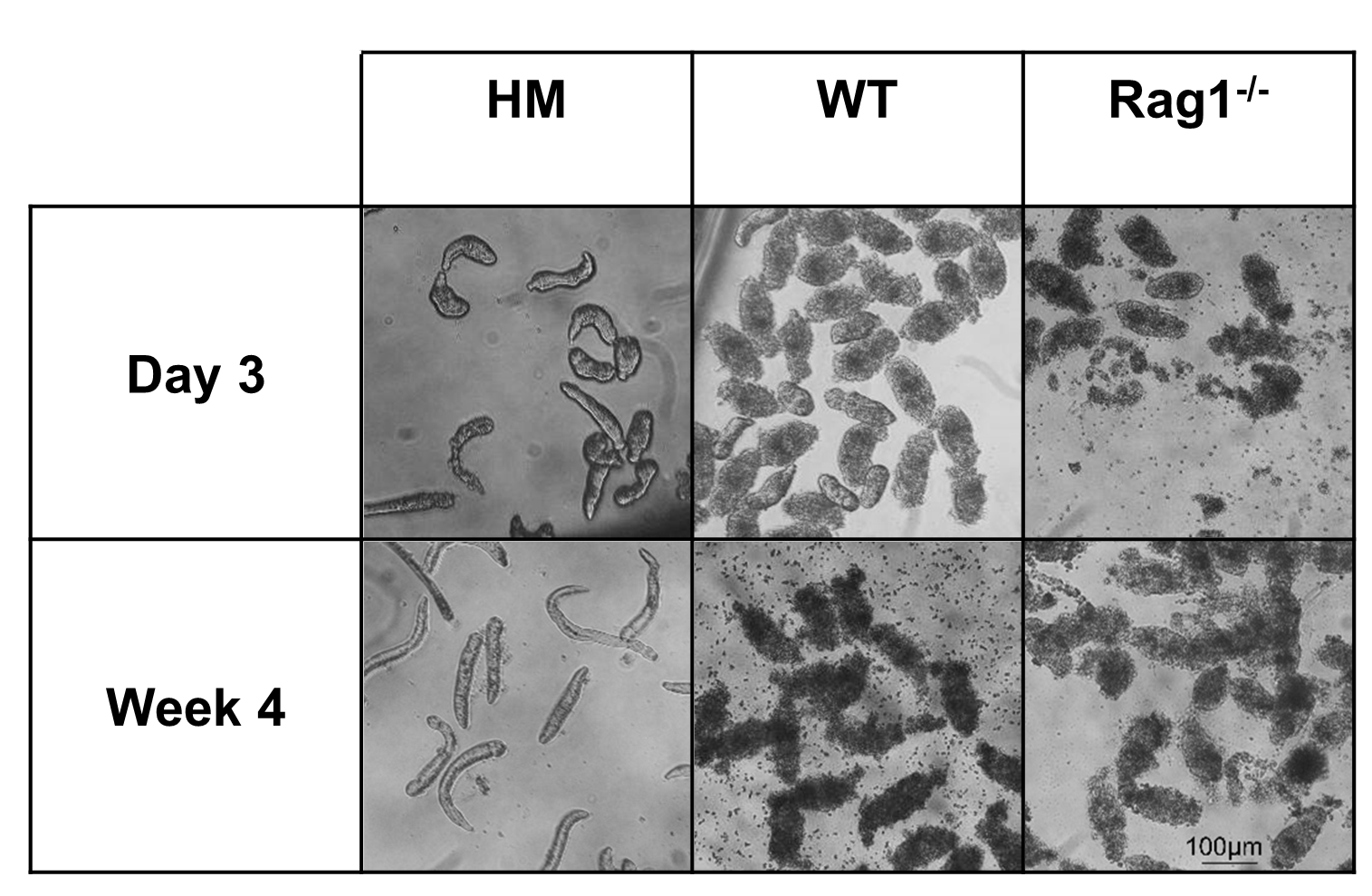

Supplement: Supplementary Figure 1 — Morphological effect of Rag1-/- serum on NTS. NTS were cultured in HM in the presence or absence of 20% WT or Rag1-/- serum. Microphotographs were taken at indicated time points with a digital camera fitted to an inverted microscope (10x). [file Image_1.tif]

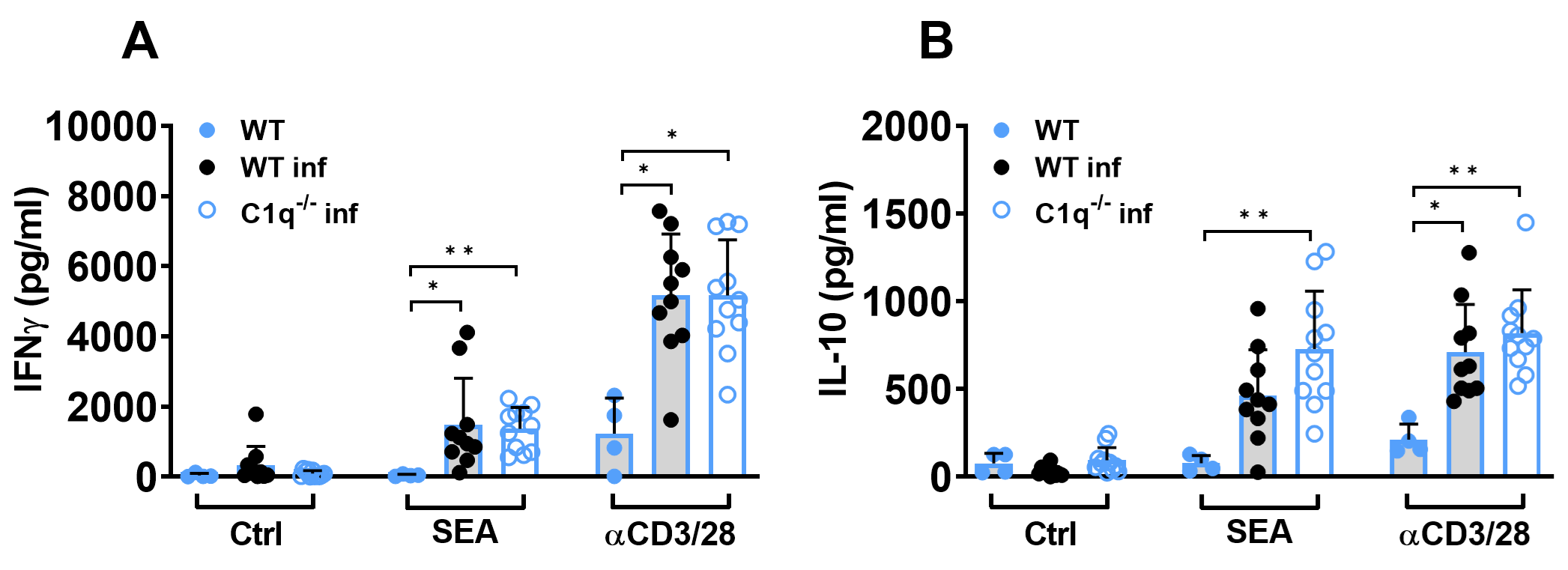

Supplement: Supplementary Figure 2 — Loss of C1q does not alter cytokine production by splenocytes. Lymphocytes (2x10^5^) from wild-type or C1q-/- mice infected for 11 weeks with S. mansoni were re-stimulated in vitro with or without (Ctrl) SEA (20 µg/ml) or anti-CD3/28 (1µg/ml) for 48 h. Secreted levels of IFN-γ (A) or IL-10 (B) were analyzed in the culture supernatants by ELISA. Graph shows representative data from at least 3 independent infection experiments. Each data point has been shown as mean ± SD of at least three technical replicates. (*P <0.05, **P <0.01). Inf, infected. [file Image_2.tif]
