# Supplementary material for: Host-Specific Serum Factors Control the Development and Survival of Schistosoma mansoni
Source: Front Immunol. 2021 Apr 23;12:635622. doi: 10.3389/fimmu.2021.635622 (PMC8103320; doi:10.3389/fimmu.2021.635622)
Supplement: Supplementary Table 1 — Viability scoring points description. [file Table_1.pdf]

Viability score points		0	1	2	3
Description of parameter	motility	Regular, non-hectic, with change in direction	Regular, reduced activity	Irregular and hectic or only internal gut movements	No movement
	morphology	Sharp outline, no blebbing	Sharp outline	Blebbing of the tegument	Blebbing, rough tegument, blurred contour
	granularity	No granularity	Light granularity	Moderate granularity	Heavy granularity
